# Fibrosis and Perinatal Features Correlated with Telomere Shortening in Pediatric Metabolic Dysfunction-Associated Steatotic Liver Disease

**DOI:** 10.3390/life16071068

**Published:** 2026-06-26

**Authors:** Maria Rita Braghini, Salvatore Daniele Bianco, Marzia Bianchi, Giulia Andolina, Antonella Mosca, Cristiano De Stefanis, Michela Piccione, Paola Francalanci, Clara Balsano, Luca Miele, Tommaso Mazza, Anna Alisi

**Affiliations:** 1 Research Unit of Genetics of Complex Phenotypes, Bambino Gesù Children’s Hospital, IRCCS, 00146 Rome, Italy; mariarita.braghini@opbg.net (M.R.B.); marzia.bianchi@opbg.net (M.B.); giulia.andolina@opbg.net (G.A.); 2 Laboratory of Computational Biology and Bioinformatics UOS, Fondazione Policlinico Universitario A. Gemelli, IRCCS, 00168 Rome, Italy; salvatoredaniele.bianco@guest.policlinicogemelli.it (S.D.B.); tommaso.mazza@policlinicogemelli.it (T.M.); 3 Division of Metabolic Diseases and Hepatology, Bambino Gesù Children’s Hospital, IRCCS, 00165 Rome, Italy; antonella.mosca@opbg.net; 4 Core Facilities, Bambino Gesù Children’s Hospital, IRCCS, 00146 Rome, Italy; cristiano.destefanis@opbg.net (C.D.S.); michela.piccione@opbg.net (M.P.); 5 Molecular Pathology Research Unit, Bambino Gesù Children’s Hospital, IRCCS, 00165 Rome, Italy; paola.francalanci@opbg.net; 6 Geriatric Unit, Department of Life, Health and Environmental Sciences-MESVA, School of Emergency and Urgency Medicine, University of L’Aquila, 67100 L’Aquila, Italy; clara.balsano@univaq.it; 7 Francesco Balsano Foundation, Via Giovanni Battista Martini 6, 00198 Rome, Italy; 8 CEMAD Digestive Diseases Center, Fondazione Policlinico Universitario “A. Gemelli” IRCCS, Università Cattolica del Sacro Cuore, 00168 Rome, Italy; luca.miele@policlinicogemelli.it

**Keywords:** MASLD, MASH, fibrosis, telomeres, children

## Abstract

Metabolic dysfunction-associated steatotic liver disease (MASLD) is an increasingly prevalent condition in both adults and children. Dysregulated telomere maintenance has been proposed as a mechanism underlying disease progression, although pediatric evidence remains limited and controversial. This study aimed to investigate the relationship between telomere length (TL) and hepato-metabolic features in children with MASLD. A total of 212 pediatric patients with biopsy-proven MASLD and 40 controls were enrolled. Telomere length in leukocytes (LTL) and liver tissue (HTL) was measured using quantitative polymerase chain reaction, and telomerase reverse transcriptase (TERT) mRNA and protein expression were also evaluated. Associations between TL and clinical, metabolic, and perinatal variables were analyzed. Children with MASLD showed significantly shorter LTL and HTL compared to controls. Shorter LTL was observed in more advanced steatohepatitis (MASH) and was associated with fibrosis severity. TERT expression was reduced in patients. LTL was also associated with perinatal factors, including preterm birth and low birthweight. Multivariable analysis identified MASH, fibrosis, and small-for-gestational-age status as independently associated with shorter LTL. In conclusion, LTL is associated with disease severity in pediatric MASLD, particularly fibrosis. These findings support a potential role of telomere dynamics in disease progression, although causal relationships require confirmation in longitudinal studies.

## 1. Introduction

The recently established definitions of metabolic dysfunction-associated steatotic liver disease (MASLD) and metabolic dysfunction-associated steatohepatitis (MASH), which replaced the term non-alcoholic fatty liver disease (NAFLD), align more closely with the underlying cardiometabolic mechanisms that accompany liver steatosis in both adult and pediatric populations [1,2].

The global prevalence of MASLD among adults is around 38% in the general population, rising to 60% among individuals who are obese and/or have type 2 diabetes mellitus (T2DM). Meanwhile, 7–14% of children and adolescents have MASLD [3,4]. The increased prevalence of MASLD is therefore a real concern, and although recent clinical trials have identified some drugs for adults that could reduce this escalation in the coming years, the complications associated with this multifaceted disease highlight the need for a more holistic approach [3,5,6].

In addition to the well-known genetic susceptibility, an increasing number of studies have highlighted the role of epigenetics in the complex inheritance of MASLD [7]. Indeed, several prenatal and in utero epigenetic processes have been reported to be associated with disease susceptibility by producing long-term changes in gene transcription [8]. Moreover, the association among genetic/epigenetic inheritance, lifestyle, and environmental exposure can define patterns that contribute to MASLD development and progression by altering transcriptional networks implicated in redox and lipid homeostasis, peroxisome and mitochondrial function, inflammation, and cellular senescence [9,10,11]. This latter mechanism, recognized as a key contributor to age-related adverse MASLD progression, is driven by multiple factors, including telomere attrition and DNA methylation [12,13]. Telomere length (TL) is a hereditary trait regulated by the action of a ribonucleoprotein complex consisting of a TERT catalytic subunit that synthesizes new telomeric repeats by copying its RNA component [14,15]. Telomere attrition is the shortening of telomeres that naturally occurs with each cell division. In fact, during DNA replication, owing to the mechanism of action of DNA polymerase, the length of the DNA is lost from the 5′ end of the chromosome. However, telomere attrition is also a hallmark of aging associated with obesity and several cardiometabolic diseases in adult populations yet remains poorly investigated in MASLD [13,16]. Clinical studies in adults have found an association between shorter leukocyte TL (LTL) and the prevalence of MASLD [17,18,19,20], while a few studies have also shown that TL is associated with liver damage [18,21,22]. However, clear evidence of the correlation between LTL shortening and MASLD in children remains lacking.

Here, we first analyzed LTL in 212 children with biopsy-proven MASLD, compared with 40 children with a healthy liver, and then evaluated the potential association between LTL and anthropological, clinical, histological, and perinatal features in pediatric MASLD.

## 2. Materials and Methods

### 2.1. Patients

This study included 212 pediatric patients with biopsy-proven MASLD and 40 age- and sex-matched control children. Control samples were obtained from non-obese pediatric patients without clinical or biochemical evidence of liver disease who were undergoing routine clinical testing. Residual blood specimens were collected at the end of the testing process and stored at −80 °C until processing. Patients with MASLD were enrolled at the Hepatology Unit of the Bambino Gesù Children’s Hospital from March 2019 to April 2023, while patients’ samples and data were collected at the Research Unit of Genetics of Complex Phenotypes. For all patients, clinical data of height, weight, waist circumference, and body mass index (BMI) were recorded at the time of enrollment using standard procedures. Metabolic variables, including triglycerides, total cholesterol, high-density lipoprotein (HDL) cholesterol, low-density lipoprotein (LDL) cholesterol, alanine aminotransferase (ALT), aspartate aminotransferase (AST), and gamma-glutamyl transpeptidase (GGT) levels, were measured using standard laboratory methods at enrollment. The homeostasis model assessment of insulin resistance (HOMA-IR) was calculated using the following formula: fasting insulin x fasting glucose/22.5. The study was conducted in accordance with the principles of the Declaration of Helsinki and approved by the Ethics Committee of Bambino Gesù Children’s Hospital. Written informed consent was obtained from the child’s parents or legal guardians.

### 2.2. Liver Histology

Liver biopsies were performed in all patients using an automatic core biopsy needle (16 or 18 gauge) under general anesthesia and ultrasound guidance. Histological evaluation and grading of fibrosis were performed according to the NASH-Clinical Research Network CRN criteria [23]. Diagnosis of MASH was defined according to the algorithm recently suggested in the Delphi consensus [1]. In particular, MASH was defined with a NAS ≥ 5, or with a NAS ≥ 4 plus fibrosis F > 1. A definitive diagnosis of MASH or non-MASH was reached only when the two pathologists agreed on the diagnosis. The two pathologists who performed the histological evaluation were blinded to TL assessment results.

### 2.3. Assessment of TL

For the assessment of LTL, DNA was extracted from peripheral blood using the QIAmp DNA Blood Mini Kit (Qiagen, Hilden, Germany), while for the assessment of HTL, DNA was extracted from liver tissues using the AllPrep DNA/RNA/Protein Mini Kit (Qiagen). DNA concentration and purity were assessed using a NanoDrop spectrophotometer (Thermo Fisher Scientific, Waltham, MA, USA), ensuring an A260/A280 ratio between 1.8 and 2.0, and samples were stored at −80 °C until TL assessment. Next, TL was determined using the real-time quantitative polymerase chain reaction (qPCR) method, as reported by O’Callaghan and Fenech [24]. In particular, a qPCR assay was performed by a QuantStudio 7 Pro Real-Time PCR System (Applied Biosystems-Thermo Fisher Scientific) using the following primers: Telomere forward (Fwd) (CGGTTTGTTTGGGTTTGGGTTTGGGTTTGGGTTTGGGTT) and Telomere reverse (Rev) (GGCTTGCCTTACCCTTACCCTTACCCTTACCCTTACCCT). To normalize DNA input, the gene 36B4 (acid ribosomal protein 36B4) was used as a single-copy gene and was measured using the following primers: 36B4 Fwd (CAGCAAGTGGGAAGGTGTAATCC) and 36B4 Rev (CCCATTCTATCATCAACGGGTACAA). The measure of telomeres and single-copy genes was determined by generating a standard curve on each plate by performing a serial dilution of a known amount of oligomer standard for either telomere (TTAGGG)14 or the 36B4 gene (CAGCAAGTGGGAAGGTGTAATCCGTCTCCACAGACAAGGCCAGGACTCGTTTGTACCCGTTGATGATAGAATGGG). All primers were used at a final concentration of 0.1 μM and were purchased from Merck (Rahway, NJ, USA).

### 2.4. Nucleic Acid Extraction and qPCR in Liver Tissue Samples

Total RNA and DNA were extracted from liver specimens using the AllPrep DNA/RNA/Protein Mini Kit (Qiagen) according to the manufacturer’s protocol. cDNA reverse transcription was conducted using the SuperScript VILO cDNA Synthesis Kit (Invitrogen-Thermo Fisher Scientific). qPCR amplification, detection, and analysis were performed by the QuantStudio 7 Pro Real-Time PCR System (Applied Biosystems-Thermo Fisher Scientific) using TaqMan Universal PCR Master Mix and No AmpErase UNG (Applied Biosystems-Thermo Fisher Scientific). The mRNA expression level of the TERT gene was determined using a specific TaqMan probe (TERT, Hs00972650_m1) and normalized to the endogenous control gene glyceraldehyde-3-phosphate dehydrogenase (GAPDH, Hs02786624_g1) purchased from Applied Biosystems-Thermo Fisher Scientific. Gene expression levels were represented as fold-changes relative to the control and were calculated using the ΔΔCt method.

### 2.5. Immunofluorescence in Liver Tissue Samples

The 2 µm slices were obtained from formalin-fixed and paraffin-embedded human liver tissue specimens. After dewaxing and rehydration, heat-induced epitope retrieval was performed by boiling the slides with Dako Target Retrieval Solution EDTA (pH 9) (Dako-Agilent Technologies, Santa Clara, CA, USA). The primary antibody was diluted 1:100 in PBS/1% BSA and incubated overnight at 4 °C (TERT antibody, ab230527, Abcam, Cambridge, UK). The primary antibody was revealed with the secondary antibody diluted 1:500 in PBS/1% BSA (Rabbit IgG (H+L) Cross-Adsorbed Secondary Antibody Alexa Fluor 488, A-11070, Invitrogen-Thermo Fisher Scientific). Cell nuclei were counterstained with Hoechst (Invitrogen-Thermo Fisher Scientific). Samples were digitalized by using the Hamamatsu Nanozoomer S60 digital slide scanner C13210–01 (Hamamatsu Photonics, Shizuoka, Japan), equipped with Olympus 20×/0.75 and 40×/1.40 PlanSApo objectives (Olympus, Tokyo, Japan), a fluorescence imaging module with an LX2000 mercury lamp (Hamamatsu Photonics), and a linear ORCA-Flash 4.0 digital CMOS camera (Hamamatsu Photonics). Whole-slide images were used to manually draw the region of interest and perform quantitative fluorescence imaging analysis (QFIA). The average of fluorescence intensity in at least five fields per sample was calculated using ImageJ software, version 1.54n (National Institutes of Health, Bethesda, MD, USA). Images were also acquired by confocal microscopy performed on an Olympus Fluoview FV3000 Confocal Laser Scanning Microscope (Olympus).

### 2.6. Sample Size Estimation

Assuming a mean clinically relevant difference of 0.07 kb between the control and MASLD groups, based on data previously reported for young adults by Kim et al. [18], the estimated minimum sample size was 26 subjects per group using a two-sided significance level (α) of 0.05 and a statistical power of 95%. For the comparison between MASH and non-MASH, the estimate was of at least 66 subjects per group using a two-sided significance level (α) of 0.05 and a statistical power of 85%.

### 2.7. Statistics

Differences between groups were assessed using the Mann–Whitney U test for comparisons between two independent groups when variables were not normally distributed, or one-way ANOVA with Tukey’s correction for comparisons among more than two groups when normality assumptions were satisfied. Associations between telomere length (TL) and clinical or perinatal variables were evaluated using Spearman’s rank correlation. These analyses were performed using GraphPad Prism version 8.4.3 (GraphPad Software, La Jolla, CA, USA). Multiple linear regression analyses were subsequently performed to evaluate the independent relationship between log-transformed TL and anthropometric, biochemical, and histological variables. Prior to model fitting, missing data were addressed using mean imputation for continuous variables and mode imputation for categorical variables. The models assumed normally distributed errors and were fitted using the ordinary least squares (OLS) method. Multicollinearity among predictors was assessed by variance inflation factors (VIF). These analyses were conducted using Python version 3.12 in Google Colab with the Pandas (v2.2.3) and Statsmodels (v0.14.4) libraries. To further assess the association between TL and categorical predictors such as fibrosis stage, portal inflammation, and perinatal growth categories (SGA, AGA, LGA), a generalized linear model (GLM) was also fitted with TL as the dependent variable. All the multivariable models were adjusted for age and sex as potential confounders. Coefficients from each prespecified multivariable regression model were evaluated using the corresponding t-statistics, equivalent to Wald tests for individual regression coefficients. A two-sided p-value <0.05 was considered statistically significant.

## 3. Results

### 3.1. LTL in Children and Adolescents with MASLD

**Figure 1:**
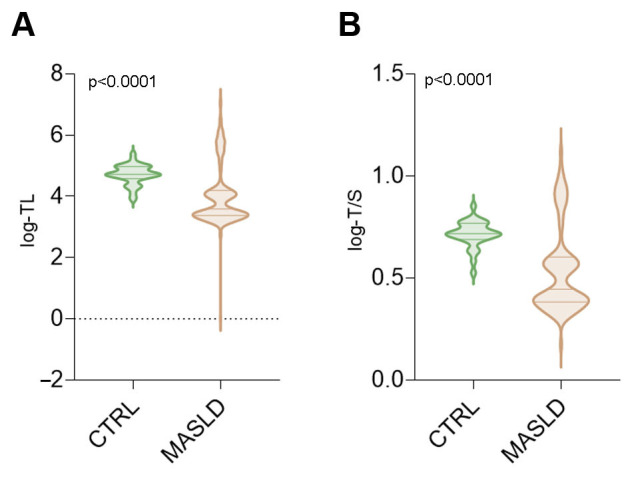
LTL was lower in pediatric patients with MASLD compared with controls. Violin plots reporting the mean log-TL (**A**) and log-T/S (**B**) in CTRL and in patients with MASLD. Mann–Whitney U test: *p* < 0.0001.

The study included 212 patients with biopsy-proven MASLD, comprising 131 males and 81 females, with a median age of 13.7 years (range, 5.2–17.9 years) and 40 control children (CTRL), comprising 21 males and 19 females, with a median age of 12 years (range, 5–18 years). Children with MASLD exhibited significantly lower HDL cholesterol and higher BMI, triglyceride, ALT, and AST levels compared to the control group (*p* < 0.0001). No significant differences were observed between groups with respect to age and sex distribution. The anthropometric and biochemical characteristics of the study population are reported in Table S1. We assessed LTL across all patient groups and found it was significantly shorter in patients with MASLD than in CTRL (*p* < 0.001). Figure 1A,B shows LTL reported either as the natural logarithm of TL in kb per human diploid genome (log-TL) or as the natural logarithm of telomere/single copy gene ratio (log-T/S). As the values of LTL expressed in kb or as T/S ratio followed the same trend, in the following analyses we only show LTL expressed as the natural logarithm of TL in kb per human diploid genome (log-TL).

### 3.2. LTL in Pediatric MASLD Stratified for the Presence of MASH

According to the Delphi consensus guidelines for the diagnosis of MASH [1], our cohort of patients with MASLD was divided into non-MASH (67 patients, 31.6%) and MASH (145 patients, 68.4%) groups. Biochemical and anthropometric variables of the patients are shown in Table 1. Briefly, patients with MASH had significantly higher (*p* < 0.0001) levels of ALT, AST, GGT, and HOMA-IR and significantly lower (*p* = 0.0001) levels of LDL cholesterol than those without MASH. The histological characteristics of the MASH and non-MASH groups are presented in Table S2. As shown, patients with MASH were selected by fibrosis stages that were adequately represented within the cohort (F0-F2), preventing meaningful statistical analyses.

**Figure 2:**
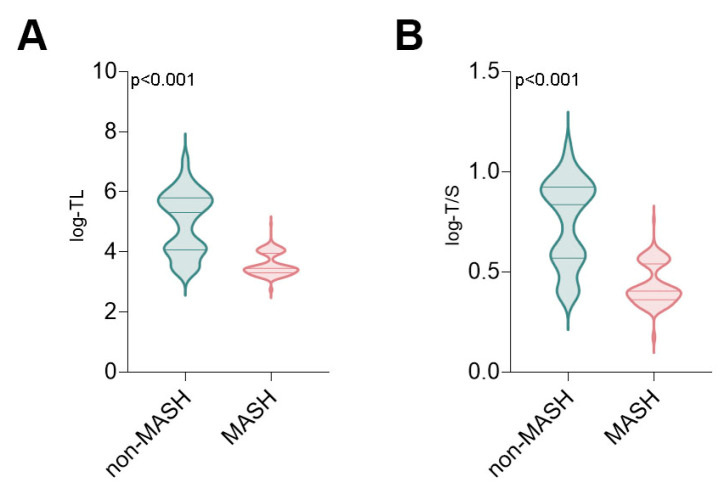
Evaluation of LTL in pediatric patients with MASLD, non-MASH, and MASH. Violin plots reporting the (**A**) mean log-TL and (**B**) log-T/S in patients with MASLD, non-MASH, and MASH. Mann–Whitney U test: *p* < 0.001.

We analyzed the differences in LTL in these groups, and it emerged that patients with MASH had significantly shorter LTL than non-MASH patients (*p* < 0.001) (Figure 2A,B). Spearman’s correlation analysis confirmed an inverse association between the presence of MASH and LTL (r = −0.64, *p* < 0.001).

### 3.3. LTL Correlation with Anthropometric, Metabolic, and Histological Parameters in Patients with MASLD

**Figure 3:**
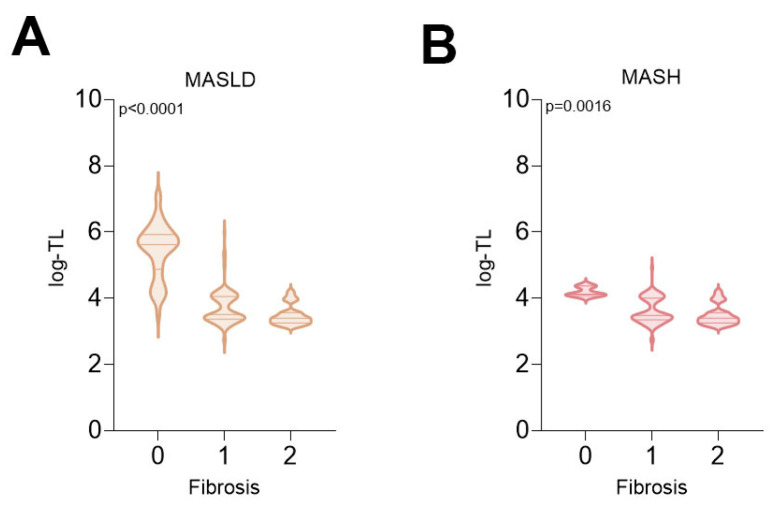
LTL is associated with liver fibrosis grade. Violin plots reporting the mean log-TL in (**A**) all patients with MASLD and in (**B**) the subgroup of patients with MASH and fibrosis grades 0, 1, and 2. One-way ANOVA test with Tukey correction.

To further investigate the significance of the LTL decrease in patients with MASH, we conducted a multiple linear regression analysis considering log-TL as the dependent variable and anthropometric and biochemical parameters as independent variables. As shown in Table S3, multiple linear regression analysis revealed that neither anthropometric nor biochemical features had a significant impact on LTL. Instead, when we used histological features (i.e., steatosis, portal inflammation, lobular inflammation, ballooning, and fibrosis) as independent variables, we found that LTL was significantly correlated with fibrosis (*p* < 0.001) (Table 2). Indeed, LTL significantly decreased with increasing fibrosis severity in MASLD patients (F_2, 209_ = 188.8; *p* < 0.0001) (Figure 3A). Among all patients with MASLD, those with fibrosis grade 0 had a mean log-TL of 5.44, those with fibrosis grade 1 had a mean log-TL of 3.72, and those with fibrosis grade 2 had a mean log-TL of 3.483 (Figure 3A). The same significant decreasing trend (F_2, 142_ = 6.744; *p* = 0.0016) was observed in the subgroup of patients with MASH, with mean log-TL values of 4.19, 3.611, and 3.483 for F0, F1, and F2, respectively (Figure 3B).

### 3.4. TL Evaluation in the Liver of Patients with MASLD

**Figure 4:**
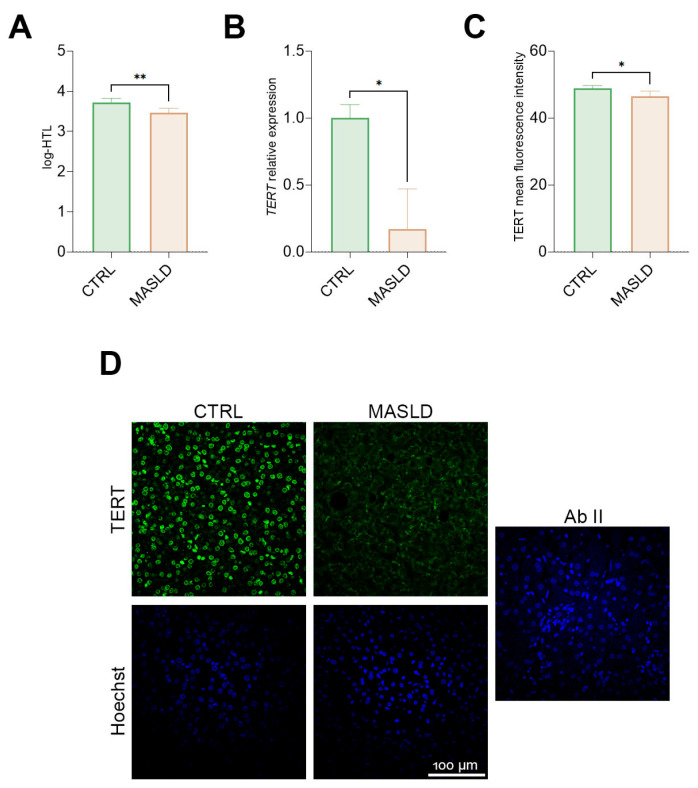
HTL and TERT expression in the liver of patients with MASLD. Histograms reporting the (**A**) mean values of log-HTL, (**B**) TERT mRNA relative expression, and (**C**) TERT mean fluorescence intensity in CTRL and MASLD groups. Mann–Whitney U test: * *p* < 0.05, ** *p* < 0.01. (**D**) Representative immunofluorescence by confocal imaging of TERT protein in CTRL and MASLD. The technical control of secondary antibody staining is reported (Ab II). Images were taken at 40× magnification.

Next, we analyzed HTL in a sub-cohort of 15 patients with MASLD (MASLD) for whom liver biopsy tissue was available in our laboratory and three healthy liver donors (CTRL). We found that the mean log-HTL of patients with MASLD was 3.463, while that of controls was 3.725 (*p* = 0.002) (Figure 4A). To further investigate the molecular mechanisms underlying telomere shortening, we analyzed the expression of the enzyme TERT responsible for telomere maintenance. Interestingly, qPCR analysis revealed a decrease in *TERT* gene (Figure 4B) and protein expression in the MASLD group compared to the CTRL group (Figure 4C,D). Finally, despite the very limited sample size, an explorative multiple linear regression analysis, with log-HTL as the dependent variable and the histological features of steatosis, portal inflammation, lobular inflammation, ballooning, fibrosis, and NAS score as independent variables, was performed. The preliminary results suggested an association between fibrosis and log-HTL (SE = 0.064, β = –0.169, R^2^ = 0.605, *p* = 0.037), with portal inflammation also correlated with log-HTL (SE = 0.100, β = −0.265, R^2^ = 0.605, *p* = 0.038).

### 3.5. LTL Association with Perinatal Features Data of Patients with MASLD

**Figure 5:**
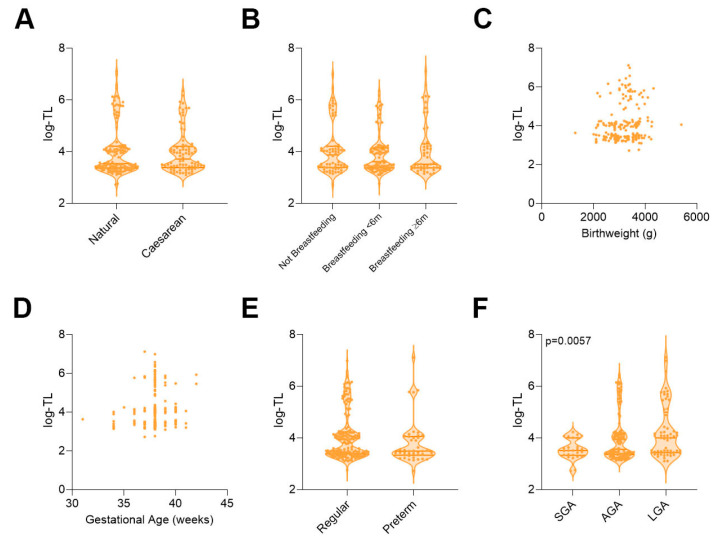
LTL association with perinatal features data of patients with MASLD. Violin and scatter plots reporting the values of log-TL in patients with MASLD in relation to patients’ mode of delivery (**A**), breastfeeding status (**B**), birthweight (**C**), gestational age (**D**), and preterm status (**E**) and grouped for being born SGA, AGA, and LGA (**F**). One-way ANOVA test with Tukey correction for comparison between SGA/AGA/LGA groups.

The relationship between MASLD-related damage and LTL in an adult setting may be attributed to chronic low-grade inflammation, multiple tissue inflammation, and lymphocyte senescence [20]. However, it remains to be explained why LTL shortening also occurs in pediatric MASLD. One hypothesis is that TL dynamics during childhood could be predetermined before birth by early-life factors, as suggested by previous studies [25,26]. Therefore, we investigated the possible association between LTL and early-life factors, including mode of delivery (natural or cesarean) (Figure 5A); breastfeeding status (not, less than, or more than 6 months) (Figure 5B); birth weight (grams) (Figure 5C); gestational age (weeks) (Figure 5D); and preterm status (Figure 5E) in our cohort of patients with MASLD. Interestingly, Spearman correlation analysis revealed that LTL was significantly associated with birthweight (r = 0.163, *p* = 0.017), gestational age (r = 0.138, *p* = 0.044), and preterm birth (r = −0.169, *p* = 0.014). Therefore, to further investigate the relationship between LTL, birthweight, gestational age, and MASLD pattern, the patients were divided into three groups based on whether they were born with a weight that was small, appropriate, or large for their gestational age (SGA, AGA, or LGA). Interestingly, multiple comparisons between the groups revealed significant differences in log-TL between patients born SGA and LGA (F_2, 209_ = 5.304; *p* = 0.0057) (Figure 5F).

### 3.6. Construction of a Generalized Linear Model with Emerging Histological and Perinatal Features as Predictors of LTL

**Figure 6:**
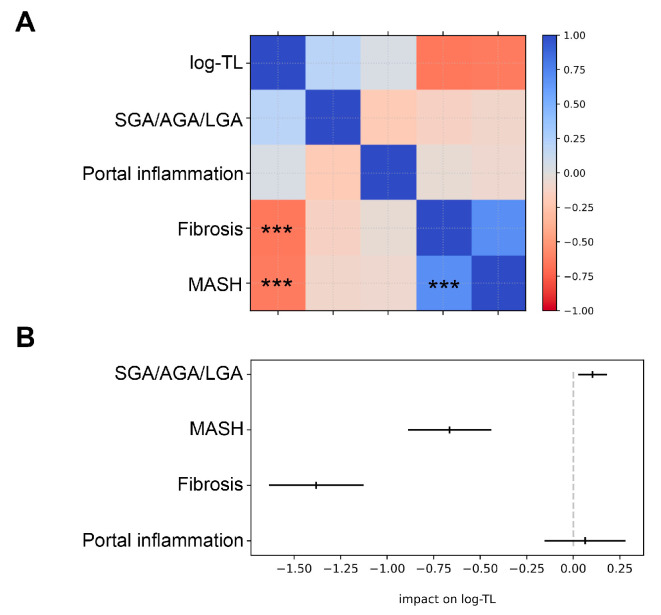
GLM of LTL according to histological and perinatal features in patients with MASLD. (**A**) Pairwise Spearman correlations between clinical variables. *** *p* < 0.001 (**B**) Point estimates (vertical ticks) and 95% confidence intervals (horizontal lines) represent the coefficients from a generalized linear model (GLM) fitted to log-TL as a function of the following clinical variables: Small/Appropriate/Large for Gestational Age (continuous), Portal Inflammation, Fibrosis, and MASH (all categorical). A vertical dashed line at 0 indicates no effect on log-TL. Model adjusted for age and sex as potential confounders.

Lastly, we evaluated the relationship between the presence of fibrosis, portal inflammation, MASH, and SGA/AGA/LGA pattern with log-TL. Pairwise Spearman correlation analysis showed that log-TL was significantly inversely correlated with both fibrosis (r = −0.66, *p* = 2.06 × 10^−27^) and MASH (r = −0.64, *p* = 1.69 × 10^−25^). As expected, fibrosis and MASH were positively correlated (r = 0.69, *p* = 3.27 × 10^−31^) (Figure 6A). Finally, we fitted a generalized linear model (GLM) with log-TL as the dependent variable and fibrosis, MASH, portal inflammation, and SGA/AGA/LGA classification as predictors. GLM showed a 0.89 pseudo-R^2^. Both fibrosis and MASH were significantly negatively associated with log-TL (Wald tests: *p* < 0.001), while being born LGA was significantly associated with higher log-TL values (Wald test: *p* = 0.019). No significant association was observed for portal inflammation (Wald test: *p* = 0.557) (Figure 6B). Detailed GLM coefficients are reported in Table S4.

## 4. Discussion

In this study, we evaluated TL in peripheral blood leukocytes and liver tissue from a cohort of pediatric patients with biopsy-proven MASLD, compared with healthy controls. To date, a few studies have analyzed TL in children and adolescents with MASLD; however, available data remain controversial [27,28,29]. Our results demonstrated that LTL was lower in patients with MASLD than in healthy controls. Notably, shorter LTL was observed in patients with more advanced liver damage, with MASH patients showing significantly reduced LTL compared to those in earlier disease stages (non-MASH). These findings are consistent with previous studies in adult MASLD populations, which have reported associations between telomere shortening and disease severity [20,30,31]. In addition, several adult studies have demonstrated that shorter telomeres are associated with more advanced fibrosis stages [18,22,32,33]. Accordingly, we found a significant negative correlation between fibrosis grade, as evaluated by biopsy, and LTL, with progressively shorter telomeres observed at increasing fibrosis stages. Interestingly, Wojcicki et al. [28] reported a different pattern in a pediatric MASLD cohort. Their findings suggested an association between longer LTL and inflammatory features, particularly lobular inflammation, whereas no significant association with fibrosis was observed. This discrepancy with our findings and other studies in adults may reflect differences in study design, population characteristics, disease stage, or methodological approaches and further highlights the complexity of telomere biology in pediatric MASLD that may involve complex compensatory mechanisms. Further longitudinal studies are required to clarify the biological role of telomere alterations in pediatric MASLD.

Consistent with leukocyte data, we found shorter TL in the livers of patients with MASLD than in healthy donors. Our preliminary, explorative multivariable analysis suggested a negative correlation between HTL, fibrosis grade, and severity of portal inflammation, a hallmark of necro-inflammatory activity in pediatric MASLD [34]. To explore potential mechanisms underlying telomere shortening, we assessed TERT gene and protein expression in a sub-cohort of liver samples. Patients with MASLD showed significantly reduced hepatic TERT mRNA and protein levels compared with controls. Previous studies have shown that TERT deficiency or genetic mutations may accelerate telomere shortening and predisposition to liver disease progression [35,36]. In line with this, Donati et al. [37] reported progressive LTL shortening across stages from healthy liver to cirrhosis and hepatocellular carcinoma. Although we cannot exclude the contribution of constitutional genetic factors, it is more plausible that LTL erosion results from the progression of chronic liver disease [38]. Epigenetic regulation and the so-called “telomere position effect” [39] may represent potential mechanisms underlying TERT downregulation and telomere shortening in MASLD. Although exploratory and based on a limited sample size, these findings suggest a potential association between telomere shortening and liver disease severity, warranting further investigation in larger, independent cohorts.

While telomere attrition is attenuated during childhood and adolescence, early-life factors may interfere with TL regulation in pediatric MASLD. Factors such as birthweight, gestational age, delivery mode, and breastfeeding have been suggested to influence both TL and disease severity [8,40,41]. In our cohort, LTL was significantly associated with preterm birth and birthweight. Importantly, fibrosis, MASH, and being born small for gestational age (SGA) were associated with lower LTL. These findings support the hypothesis that intrauterine conditions may induce epigenetic adaptations that influence telomere dynamics from birth, potentially increasing susceptibility to MASLD and its progression later in life, as also sustained by Kim et al. [42].

This study has several important limitations. First, there is a risk of overstated causal inference. Although associations between telomere shortening and fibrosis are described, the cross-sectional design does not allow determination of temporal or causal relationships. Therefore, it cannot be established whether telomere shortening contributes to fibrosis development or is a consequence of disease progression. Second, the analysis does not fully account for potential confounding factors known to influence telomere length, such as genetic background, parental telomere length, and environmental or lifestyle exposures. This may result in residual confounding and could partially explain the observed associations. Third, although our cohort of 212 children with biopsy-proven MASLD represents a good dataset in this field, it may still be considered medium-sized for highly adjusted multivariable regression models; also, the relatively small size of the control group and the lack of an independent validation cohort may limit the generalizability of the findings. Overall, these limitations indicate that the results should be interpreted with caution and considered primarily hypothesis-generating, warranting confirmation in larger and longitudinal pediatric studies.

## 5. Conclusions

In conclusion, our study demonstrates an association between telomere dynamics and hepatic damage in pediatric MASLD, particularly with MASH and fibrosis. These findings may help identify children at higher risk of disease progression, although this potential application requires validation in longitudinal studies.

## Figures and Tables

**Table 1:** Biochemical and anthropometrical characteristics of pediatric patients with MASLD without or with MASH.

Variable	non-MASH (n = 67)	MASH (n = 145)	*p* Value
Gender (M/F)	36/31	95/50	0.1282
Age (years)	13 (5–18)	14 (6–18)	0.5834
BMI (kg/m^2^)	28.2 (16.5–47.4)	29.1 (21.2–39.9)	0.5842
WC (cm)	89 (67–134)	86 (60–112)	0.5468
Triglycerides (mg/dL)	100 (39–225)	89 (35–277)	0.2038
Total cholesterol (mg/dL)	154 (93–210)	156 (91–298)	0.5044
HDL cholesterol (mg/dL)	47 (21–114)	48 (20–112)	0.5685
LDL cholesterol (mg/dL)	97 (48–153)	73 (33–145)	0.0001
ALT (IU/mL)	25 (8–82)	40 (16–265)	<0.0001
AST (IU/mL)	25 (12–40)	31 (19–109)	<0.0001
GGT (IU/mL)	14 (3–43)	23 (9–101)	<0.0001
HOMA-IR	3.7 (0.2–10.4)	4.2 (2.1–15.8)	<0.0001

* Values are expressed as median and range (minimum–maximum). Fisher’s exact test for gender distribution and the Mann–Whitney U test for continuous variables were applied. Abbreviations: MASH, metabolic dysfunction-associated steatohepatitis; M, males; F, females; BMI, body mass index; WC, waist circumference; HDL, high-density lipoprotein; LDL, low-density lipoprotein; ALT, alanine transaminase; AST, aspartate transaminase; GGT, gamma glutamyl transpeptidase; HOMA-IR, homeostasis model assessment of insulin resistance.

**Table 2:** Multiple linear regression analysis of LTL estimates in patients with MASLD.

Dependent Variable: log-TL	Model					
	β	SE	95% CI Lower	95% CI Upper	t	*p*
Intercept	5.390	0.294	4.811	5.969	18.351	0.000
Steatosis	−0.736	0.612	−1.943	0.471	−1.202	0.231
Portal Inflammation	0.082	0.089	−0.093	0.257	0.924	0.357
Lobular Inflammation	−0.759	0.624	−1.989	0.471	−1.217	0.225
Ballooning	−0.742	0.628	−1.981	0.496	−1.182	0.239
Fibrosis	−0.703	0.079	−0.858	−0.548	−8.953	0.000
NAS	0.509	0.616	−0.706	1.724	0.826	0.410

* Model R^2^ = 0.587. Model adjusted for age and sex as potential confounders. Abbreviations: log-TL, natural logarithm of telomere length in kilobases; β, regression coefficient; SE, standard error; 95% CI lower and 95% CI upper: lower and upper bounds of the 95% confidence interval for β; t-statistic for testing β = 0.

## Data Availability

The data that support the findings of this study are available from the corresponding author upon reasonable request.

## Supplementary Materials

The following supporting information can be downloaded at: https://www.mdpi.com/article/10.3390/life16071068/s1, Table S1: Characteristics of the study population; Table S2. Histological features of the liver of patients with MASLD. Number of cases and percentage of histological features grades in non-MASH and MASH groups; Table S3. Multiple linear regression analysis of LTL estimates in patients with MASLD; Table S4. GLM of TL according to histological and perinatal features in patients with MASLD.

## Abbreviations

The following abbreviations are used in this manuscript:

MASLD	Metabolic dysfunction-associated steatotic liver disease
TL	Telomere length
LTL	Leukocyte telomere length
HTL	Hepatic telomere length
TERT	Telomerase reverse transcriptase
MASH	Metabolic dysfunction-associated steatohepatitis
NAFLD	Non-alcoholic fatty liver disease
T2DM	Type 2 diabetes mellitus
BMI	Body mass index
HDL	High-density lipoprotein cholesterol
LDL	Low-density lipoprotein cholesterol
ALT	Alanine aminotransferase
AST	Aspartate aminotransferase
GGT	Gamma-glutamyl transpeptidase
HOMA-IR	Homeostasis model assessment of insulin resistance
qPCR	Quantitative polymerase chain reaction
QFIA	Quantitative fluorescence imaging analysis
OLS	Ordinary least squares
VIF	Variance inflation factor
GLM	Generalized linear model
log-TL	Natural logarithm of TL in kb per human diploid genome
log-T/S	Natural logarithm of telomere/single copy gene ratio
WC	Waist circumference
SGA	Small for their gestational age
AGA	Appropriate for their gestational age
LGA	Large for their gestational age
HCC	Hepatocellular carcinoma
