# Supplementary material for: Fibrosis and Perinatal Features Correlated with Telomere Shortening in Pediatric Metabolic Dysfunction-Associated Steatotic Liver Disease
Source: Life (Basel). 2026 Jun 26;16(7):1068. doi: 10.3390/life16071068 (PMC13412969; doi:10.3390/life16071068)
Supplement: Supplementary file 1 [file life-16-01068-s001.zip › life-4304976-supplementary.pdf]

Supplementary Information

Fibrosis and perinatal features correlated with telomere shortening in pediatric metabolic dysfunction-associated steatotic liver disease

Maria Rita Braghini[1], Salvatore Daniele Bianco[2], Marzia Bianchi[1], Giulia Andolina[1], Antonella Mosca[3], Cristiano De Stefanis[4], Michela Piccione[4], Paola Francalanci[5], Clara Balsano[6,7], Luca Miele[8], Tommaso Mazza[2], Anna Alisi[1,*]

List of supplementary materials:

Table S1, page 2
Table S2, page 3
Table S3, page 4
Table S4, page 5

Table S1. Characteristics of the study population.

Variable	*CTRL (n=40)*	*MASLD (n=212)*	*p value*
Gender (M/F)	21/19	131/81	0.2935
Age (years)	12 (5 – 18)	13.7 (5.2 – 17.9)	0.0799
BMI (kg/m²)	18.8 (13.1 – 25.6)	29.1 (16.5 – 47.4)	<0.0001
Triglycerides (mg/dL)	60.5 (29.0 – 128.0)	93.0 (35.0 – 277.0)	<0.0001
Total cholesterol (mg/dL)	146.0 (95.0 – 244.0)	156.0 (91.0 – 298.0)	0.2590
HDL cholesterol (mg/dL)	61.5 (32.0 – 85.0)	48.0 (20.0 – 114.0)	<0.0001
LDL cholesterol (mg/dL)	80.0 (56.0 – 160.0)	83.0 (33.0 – 153.0)	0.4944
ALT (IU/mL)	20.0 (12.0 – 59.0)	27.0 (12.0 – 109.0)	<0.0001
AST (IU/mL)	26.5 (15.0 – 43.0)	36.0 (8.0 – 265.0)	<0.0001

Values are expressed as median and range (minimum – maximum). Fisher's exact test for gender distribution and the Mann-Whitney test for continuous variables.

Abbreviations: CTRL, controls; MASLD, metabolic dysfunction-associated steatotic liver disease; M, males; F, females; BMI, body mass index; HDL, high-density lipoprotein; LDL, low-density lipoprotein; ALT, alanine transaminase; AST, aspartate transaminase.

Table S2. Histological features of the liver of patients with MASLD. Number of cases and percentage of histological features grades in non-MASH and MASH groups.

Histological traits (stages)	*non-MASH (n=67)*	*MASH (n=145)*
Steatosis		
1	47 (70%)	5 (3%)
2	18 (27%)	40 (28%)
3	2 (3%)	100 (69%)
Portal Inflammation		
0	7 (10%)	24 (17%)
1	56 (84%)	105 (72%)
2	4 (6%)	16 (11%)
Lobular Inflammation		
0	19 (29%)	-
1	47 (70%)	136 (94%)
2	1 (1%)	9 (6%)
Ballooning		
0	31 (46%)	1 (1%)
1	35 (53%)	82 (57%)
2	1 (1%)	62 (42%)
Fibrosis		
0	42 (63%)	3 (2%)
1	25 (37%)	85 (59%)
2	-	57 (39%)
3	-	-
NAS		
1	3 (4%)	-
2	24 (36%)	-
3	36 (54%)	-
4	4 (6%)	30 (21%)
5	-	64 (44%)
6	-	50 (34%)
7	-	1 (1%)

Abbreviations: MASH, metabolic dysfunction-associated steatohepatitis; NAS, NAFLD activity score.

Table S3. Multiple linear regression analysis of LTL estimates in patients with MASLD.

Dependent variable: log-TL	*Model*					
	β	*SE*	*95% CI lower*	*95% CI upper*	*t*	*p*
Intercept	3.793	0.307	3.188	4.398	12.370	0.000
Gender (M/F)	-0.004	0.046	-0.094	0.087	-0.080	0.936
Age (years)	0.000	0.008	-0.016	0.016	0.042	0.966
BMI (kg/m²)	-0.001	0.009	-0.018	0.016	-0.116	0.908
WC (cm)	0.002	0.003	-0.004	0.007	0.569	0.570
Triglycerides (mg/dL)	0.000	0.001	-0.001	0.001	-0.201	0.841
Total cholesterol (mg/dL)	-0.001	0.001	-0.002	0.001	-0.867	0.387
HDL cholesterol (mg/dL)	-0.001	0.001	-0.004	0.002	-0.516	0.606
LDL cholesterol (mg/dL)	-0.001	0.001	-0.003	0.001	-0.846	0.398
ALT (IU/mL)	0.000	0.001	-0.003	0.003	0.238	0.812
AST (IU/mL)	-0.001	0.003	-0.008	0.005	-0.436	0.663
GGT (IU/mL)	0.002	0.002	-0.001	0.005	1.161	0.247
HOMA-IR	-0.023	0.016	-0.055	0.008	-1.485	0.139

*Model R^2=0.035.

Abbreviations: log-TL, natural logarithm of telomere length in kilobases; β, regression coefficient; SE, standard error; 95% CI lower and 95% CI upper: lower and upper bounds of the 95% confidence interval for β; t-statistic for testing β = 0; M, males; F, females; BMI, body mass index; WC, waist circumference; HDL, high-density lipoprotein; LDL, low-density lipoprotein; ALT, alanine transaminase; AST, aspartate transaminase; GGT, gamma glutamyl transpeptidase; HOMA-IR, homeostasis model assessment of insulin resistance.

Table S4. GLM of TL according to histological and perinatal features in patients with MASLD.

Dependent variable: log-TL	*Model*					
	β	*SE*	*z*	*95% CI lower*	*95% CI upper*	*p*
Intercept	1.466	0.141	10.422	1.191	1.742	0.000
Portal Inflammation	0.066	0.112	0.588	-0.153	0.285	0.557
Fibrosis	-1.383	0.131	-10.591	-1.639	-1.127	0.000
MASH	-0.667	0.115	-5.800	-0.893	-0.442	0.000
SGA/AGA/LGA	0.098	0.042	2.347	0.016	0.180	0.019

*Pseudo-R^2 = 0.891. Model adjusted for age and sex as potential confounders.

Abbreviations: log-TL, natural logarithm of telomere length in kilobases; β, regression coefficient; SE, standard error; z, Wald z-statistic; 95% CI lower and 95% CI upper: lower and upper bounds of the 95% confidence interval for β; MASH, Metabolic Dysfunction-Associated Steatohepatitis; SGA/AGA/LGA, Small/Appropriate/Large for Gestational Age.
